# Effects of Crude Oil Exposure on Bioaccumulation of Polycyclic Aromatic Hydrocarbons and Survival of Adult and Larval Stages of Gelatinous Zooplankton

**DOI:** 10.1371/journal.pone.0074476

**Published:** 2013-10-07

**Authors:** Rodrigo Almeda, Zoe Wambaugh, Chao Chai, Zucheng Wang, Zhanfei Liu, Edward J. Buskey

**Affiliations:** 1 Marine Science Institute, University of Texas at Austin, Port Aransas, Texas, United States of America; 2 Department of Oceanography, Humboldt State University, Arcata, California, United States of America; 3 College of Resource and Environment, Qingdao Agricultural University, Qingdao, China; 4 School of Life Sciences, East China Normal University, Shanghai, China; Institute of Marine Research, Norway

## Abstract

Gelatinous zooplankton play an important role in marine food webs both as major consumers of metazooplankton and as prey of apex predators (e.g., tuna, sunfish, sea turtles). However, little is known about the effects of crude oil spills on these important components of planktonic communities. We determined the effects of Louisiana light sweet crude oil exposure on survival and bioaccumulation of polycyclic aromatic hydrocarbons (PAHs) in adult stages of the scyphozoans *Pelagia noctiluca* and *Aurelia aurita* and the ctenophore *Mnemiopsis leidyi*, and on survival of ephyra larvae of *A. aurita* and cydippid larvae of *M. leidyi*, in the laboratory. Adult *P. noctiluca* showed 100% mortality at oil concentration ≥20 µL L^−1^ after 16 h. In contrast, low or non-lethal effects were observed on adult stages of *A. aurita* and *M. leidyi* exposed at oil concentration ≤25 µL L^−1^ after 6 days. Survival of ephyra and cydippid larva decreased with increasing crude oil concentration and exposition time. The median lethal concentration (LC_50_) for ephyra larvae ranged from 14.41 to 0.15 µL L^−1^ after 1 and 3 days, respectively. LC_50_ for cydippid larvae ranged from 14.52 to 8.94 µL L^−1^ after 3 and 6 days, respectively. We observed selective bioaccumulation of chrysene, phenanthrene and pyrene in *A. aurita* and chrysene, pyrene, benzo[*a*]pyrene, benzo[*b*]fluoranthene, benzo[*k*]fluoranthene, and benzo[*a*]anthracene in *M. leidyi*. Overall, our results indicate that (1) *A. aurita* and *M. leidyi* adults had a high tolerance to crude oil exposure compared to other zooplankton, whereas *P. noctiluca* was highly sensitive to crude oil, (2) larval stages of gelatinous zooplankton were more sensitive to crude oil than adult stages, and (3) some of the most toxic PAHs of crude oil can be bioaccumulated in gelatinous zooplankton and potentially be transferred up the food web and contaminate apex predators.

## Introduction

Petroleum or crude oil is one of the most common pollutants released into the marine environment [1]. Rising global energy demand has resulted in an increase in the search for and transportation of crude oil in the sea, making marine environments especially susceptible to increased risk of crude oil spills [1]–[2]. Although catastrophic oil spills are not the most important source of crude oil discharge into the sea [1], [3], they have strong acute and long-term impacts on marine ecosystems, including effects from physical damages (physical contamination and smothering) and toxicity of their chemical compounds [1]. The Deepwater Horizon Oil spill in the Gulf of Mexico in 2010 is a recent example of the dramatic ecological impacts caused by oil spills in marine environments [4]–[5].

Among the biological components of marine ecosystems, planktonic organisms are particularly susceptible to crude oil pollution [6]–[8]. Zooplankton cannot overcome the effects of currents , limiting their capacity to avoid crude oil patches and, potentially, forcing them into highly polluted water masses after crude oil spills. Small crude oil droplets (1–100 µm in diameter) generated by wind and waves and or by treatment with chemical dispersants are effectively suspended in the water column [9]–[11]. These crude oil droplets, which are frequently in the food size spectra of many zooplankters, can easily interact with planktonic organisms. For instance, ingestion of crude oil droplets has been observed on different taxonomic groups of zooplankton, from micron-sized ciliates to large gelatinous zooplankton [12]–[19]. Some of the components of crude oil, such as polycyclic aromatic hydrocarbons (PAHs), can be highly toxic to zooplankton and be accumulated and transferred up through food webs [6]–[8], [20]–[21]. Therefore, given the key role of zooplankton in marine food web dynamics, biogeochemical cycling and fish recruitment [22]–[24], knowledge of the interactions between crude oil and zooplankton is crucial for our understanding of the fate of crude oil in the pelagic zone and the impact of oil spills in marine environments.

Effects of oil pollution on zooplankton vary widely depending on intrinsic (e.g., species, life stage, size) and extrinsic factors (e.g., oil concentration, exposure time, temperature, salinity, UV radiation, use of chemical dispersants) [8], [25]–[29]. Lethal and sublethal effects, including narcosis, alterations in feeding, development, and reproduction, have been frequently observed in zooplankton exposed to petroleum hydrocarbons [8], [30]–[34]. Laboratory studies have also shown that zooplankton can accumulate or metabolize certain polycyclic aromatic hydrocarbons (PAHs), suggesting that zooplankton play an important role in PAH cycling in marine environments [8], [21], [35]–[36]. However, most crude oil toxicity tests and PAH bioaccumulation studies in zooplankton has been focused on crustacean mesozooplankton and fish larvae, and little is known about the interactions between crude oil and other important components of zooplankton communities, such as gelatinous zooplankton.

Gelatinous zooplankton is a generic term used to describe a taxonomically diverse group of planktonic animals with high body water content, ≥95% (compared with 70 to 87% for crustacean zooplankton) [37]–[38]. Typically, they have soft, delicate, translucent bodies without a hard skeleton [39]. Some of the most known components of the gelatinous zooplankton are cnidarians (e.g., scyphozoans, siphonophores, “jellyfish”), ctenophores (“comb jellies”) and pelagic tunicates (e.g., appendicularians, salps, doliolids). They inhabit nearly all marine habitats, from coastal to deep waters, from tropical to polar latitudes, and may become seasonally very abundant [40]–[41]. Gelatinous zooplankton are considered to be the least understood of all zooplankton groups [42]–[43]. Their function in the marine ecosystem has been traditionally neglected or misunderstood, for example, they were considered “dead ends” of plankton food webs [43]–[46]. It has been only with the past few decades when the important role of gelatinous zooplankton in food webs and marine ecosystems has been widely recognized. Growing interest on gelatinous plankton is partly due to the perception of worldwide increases in outbreaks/blooms [40] and to the accidental introduction of certain invasive species [41], which may produce important negative ecological and socio-economic impacts (e.g. on fisheries and the tourism industry) in coastal areas [41], [47]. It is now extensively accepted that gelatinous zooplankton are key components of marine food webs both as major consumers of metazooplankton [48]–[51] and as prey of apex predators, such as tuna, billfish, sunfish and sea turtles [52]–[54]. In addition, increasing evidence has shown that gelatinous zooplankton have an influence on microbial food webs, through direct and indirect effects, and are important regulators of marine biogeochemical fluxes [55]–[58]. Gelatinous zooplankton have complex life cycles including several developmental stages with important differences in morphology, behavior and physiology [59]. For example, planktonic life-stages of many scyphozoans include adults (“medusa”) and several larval stages called “planula” and “ephyra” [59]. Most ctenophores have a tentaculate larval stage called “cydippid larva” in their life cycles [59]. However, even though larval survival is critical to adult recruitment, ecology of larvae of gelatinous zooplankton remains poorly studied in comparison to the adult phases. In the context of environmental pollution, there is an important gap in our understanding of the effects of crude oil spills on gelatinous plankton. Particularly, information is extremely scarce on the toxic effects of crude oil on developmental stages of gelatinous plankton and on the bioaccumulation of PAH in gelatinous plankton exposed to crude oil.

This study aims to investigate the toxic effects of crude oil exposure on larval and adult stages of gelatinous plankton and the bioaccumulation of PAHs in gelatinous plankton. Our specific objectives were to: (i) determine the lethal effects of different concentrations of crude oil on larvae and adults of gelatinous plankton, (ii) assess the influence of exposure time on crude oil toxicity to gelatinous plankton, and (iii) estimate the bioaccumulation of PAHs in gelatinous zooplankton. We used representative species of scyphozoans (*Pelagia noctiluca, Aurelia aurita*) and ctenophores (*Mnemiopsis leidyi*). *P. noctiluca* is typically an offshore pelagic species widely distributed in warm and temperate waters [60]. *A. aurita* is a cosmopolitan species found in a wide variety of coastal environments [60]. *M. leidyi* is a lobate ctenophore native to estuaries and coastal regions along the western Atlantic coast, and an invasive species in European coastal areas, including the North, Black, Caspian, and Mediterranean Seas, where it may regulate zooplankton communities and impact ecosystem dynamics [61]–[63]. The developmental stages used to determine the lethal effect of crude oil on larval stages of gelatinous zooplankton were ephyra larvae (*A. aurita*) and cydippid larvae (*M. leiydi*). Cydippid larvae may seasonally dominate overall abundances of ctenophore populations [64]–[65].

## Materials and Methods

### Experimental organisms

Specimens of *Pelagia noctiluca* were collected in the northern Gulf of Mexico (28° 24′ 36″N 90°512′36″W) from the R/V “Pelican” during a cruise in May 2012. Zooplankton samples were obtained by slow-speed plankton tows (10 m min^−1^) from near the bottom (50 m bottom depth) to the surface using a plankton net (50 cm diameter, 150 µm-mesh) with a 3 L plastic bag as a non-filtering cod end in order to minimize capture stress and physical damage to the organisms. Once on board, plastic bags containing the sample were kept in a cooler containing sea water at in situ temperature. In the ship's laboratory, *P. noctiluca* specimens were gently sorted from the other zooplankton using a glass beaker and placed in a container with 0.2 µm filtered sea water until experiment began (<1 h).

Adult stages of scyphozoan *Aurelia aurita* and the ctenophore *Mnemiopsis leidyi* were collected in Aransas Bay, TX (28° 05′ 01″N 96°59′30″W) in June 2012. Adult stages of the scyphozoan *A. aurita* were visually located and gently collected using an acid-washed plastic bucket. Adult stages of the ctenophore *M. leidyi* were collected from surface waters by low speed horizontal tow using a similar plankton net as used during the cruise in the northern Gulf of Mexico. Specimens were kept in large coolers filled with sea water at *in situ* temperature. In the laboratory, specimens of each species were placed in aquariums with 5 µm-filtered sea water, fed with natural zooplankton assemblages and acclimated to the laboratory conditions for 48 h.

Larval developmental stages of *Aurelia aurita* and *Mnemiopsis leidyi* were collected from the Aransas Ship Channel near the University of Texas Marine Science Institute (MSI) in Port Aransas, TX (27°49′39″N 97°4′20″W) in July 2012. Zooplankton samples from the Aransas Ship Channel were collected from surface waters by tying a microplankton net (50 µm mesh, 36 cm diameter) to the MSI pier and allowing it to stream with the tidal current for approximately 5–10 min. The plastic bags were kept in coolers filled with in situ sea water until returning to the laboratory. Cydippid larva of *M. leidyi* and ephyra stages of *A. aurita* were identified under a dissecting microscope [66], [67], gently sorted from other zooplankton with a pipette or small glass beaker, and kept in 0.2 µm filtered sea water until experiment began (within a few hours from collection).

In all cases, experimental individuals of similar size were visually sorted from the collected specimens and their average initial size was estimated from 10–20 randomly selected individuals (Table 1). The initial sizes of adults and cydippid larvae used in the experiments were estimated directly by placing the animals in shallow beakers with seawater and measuring the bell diameter using a ruler. For ephyra larvae, size was determined on fixed organisms (2% formaldehyde) under a stereomicroscope using an ocular micrometer.

**Table 1:** Characteristics of the experimental organisms (species, stage, average size) and experimental conditions concentration (Conc) of individuals per liter, number of individuals per treatment (n), temperature (T), seawater salinity (S), total exposure time, crude oil exposure levels) used in the crude oil exposure experiments.

Species	Stage	Size (cm) Avg ± SD	Conc (Ind. L^−1^)	n	T (°C)	S (‰)	Exposure time (d)	Oil exposure conc. (µL L^−1^)
*Pelagia noctiluca*	adult	1.7±0.2	5	5	22.8	33.4	<1	20, 40
*Aurelia aurita*	adult	10.8±2.5	0.1	3	24.9	33.5	6	1, 5, 25
*Aurelia aurita*	ephyra larva	0.07±0.14	25	50	25.0	33.0	3	0.1, 1, 10
*Mnemiopsis leidyi*	adult	2.7±0.4	0.7	16	24.9	33.5	6	1, 5, 25
*Mnemiopsis leidyi*	Cydippid larva	0.53±0.13	20	40	25.0	33.0	6	1, 5, 10, 25

SD: standard deviation.

No permission is required for collecting gelatinous zooplankton within state (Texas) or federal waters, unless the locations are within national parks, national seashores etc., and none of our locations were within any of these restricted areas. The University of Texas does not require an Animal Use/Animal Care protocol for invertebrates (only for vertebrates). Our studies did not involve endangered or protected species.

### Preparation of crude oil emulsions

We used Light Louisiana Sweet crude oil, which was provided by BP (BP Exploration & Production Inc.) as a surrogate for the Macondo (MC252) crude oil released in the Deepwater Horizon oil spill in the Gulf of Mexico (2010) because they are considered to have similar chemical composition and toxicity. We determined the concentration and composition of PAHs in the Light Louisiana Sweet crude oil used in the experiments.

To prepare crude oil-seawater emulsions (i.e., suspensions of oil droplets in seawater), 0.2 µm filtered seawater was placed in a glass beaker with a magnetic stir bar, which was tightly sealed with aluminum foil to prevent oil absorption on the surface of the bar. The glass beaker was placed on a magnetic stirrer plate and crude oil was added to the seawater using a Hamilton steel plunger microliter syringe. After covering the beaker with aluminum foil, the oil was emulsified by stirring at 900 rpm for 5 min at room temperature (25°C). This stir speed allowed the formation of a vortex large enough to generate oil droplets in seawater. Then, the crude oil emulsions were added to the corresponding experimental treatments at concentrations ranging from 0.1 to 40 µL L^−1^, depending on the experiment (Table 1). The formation of oil droplets was confirmed in previous tests using an Imaging Particle Analysis system (FlowCAM).

### Experimental design and procedures

Lethal effects of crude oil concentration were investigated in adult stages of *Pelagia noctiluca*, *Aurelia aurita* and *Mnemiopsis leidyi* and in *A. aurita* ephyra and *M. leidyi* cydippid larval stages. Bioaccumulation of PAHs was analyzed only in adult stages since the biomass of larval stages was not enough for reliable measurements/estimations of PAHs.

Gelatinous zooplankton were exposed to Louisiana light sweet crude oil concentrations ranging from 0.1 to 40 µL L^−1^ and incubated for 1 to 6 days depending on the species/stage in the laboratory (Table 1). Small-sized adult specimens of *Pelagia noctiluca* were incubated in 1 L beakers with 0.2 µm-filtered seawater at 23°C with artificial dim light (Table 1). Adult *Aurelia aurita* and *Mnemiopsis leidyi* were incubated in large covered aquariums (8–30 L) containing 1 µm-filtered seawater at 25°C with artificial dim light for 6 days (Table 1). To keep the oil droplets suspended in the water, turbulence in the aquariums was created by aeration using 2 glass tubes connected to an air pump. Larval stages of *A. aurita* and *M. leidyi* were incubated in polycarbonate bottles containing 0.2 µm-filtered seawater at 25°C with artificial dim light in a bench top cell production roller apparatus (Bellco Glass Inc.) at 2 rpm. All experimental and control (without oil) treatments were run simultaneously in triplicate or duplicate, except for *P. noctiluca* and *M. leidyi* experiments where one replicate per treatment was used. Seawater and crude oil were renewed every 24 hours. Adults were fed zooplankton daily with natural mesozooplankton assemblages (200–2000 µm) collected from the corresponding sampling areas. Cydippid and ephyra larvae were fed with natural microzooplankton assemblages (50–200 µm) and nauplii of the copepod *Acartia tonsa*. Natural zooplankton assemblages used as food for gelatinous zooplankton were collected daily with plankton nets. Naupliar stages of the copepod *A. tonsa* were obtained from eggs collected from a laboratory culture maintained under the conditions described in Almeda *et al.* [8]. The cryptophyte *Rhodomonas* sp. (equivalent spherical diameter, ESD = 7 µm) was added to the experimental containers to fed zooplankton. *Rhodomonas* sp. culture was grown at 24°C in 10 L glass flasks using ‘f/2’ medium.

Mortality of adults and larvae was checked every day. Adult stages and cydippid larvae, were gently placed in shallow beakers filled with 0.2 µm filtered seawater and visually checked for survival and tissue damage. In the case of ephyra larva, the contents of each bottle were gently screened through a submerged 150 µm mesh sieve, placed in glass dishes filled with 0.2 µm filtered seawater for 10 min and then, checked for larval swimming activity and survival. In most cases, dead organisms were partially or completely degraded.

After the entire incubation, adult stages of the studied species were screened through a 1000 µm mesh sieve and thoroughly rinsed with filtered seawater using a pressure sprayer to minimize oil droplets that could potentially be attached to the animals. Then, the rinsed gelatinous zooplankton were placed in covered glass flasks and frozen (−20°C) until analysis of PAHs. In addition to these final samples for PAH analysis, samples of specimens exposed to crude oil (*A. aurita* exposed to 5 and 25 µL L^−1^, and *M. leidyi* exposed to 25 µL L^−1^) for 10 min were taken to evaluate if oil droplets attached to exterior of the gelatinous zooplankton bodies may potentially affect the PAH bioaccumulation results.

### Chemical analysis

Sixteen priority PAHs defined by the US Environmental Protection Agency (EPA) were analyzed: naphthalene (Nap), acenaphthene (Ace), acenaphthylene (Acy), fluorene (Flu), phenanthrene (Phe), anthracene (An), fluoranthene (Flua), pyrene (Pyr), benzo[*a*]anthracene (BaA), chrysene (Chr), benzo[*b*]fluoranthene (BbF), benzo[*k,*j]fluoranthene (BkF), benzo[*a*]pyrene (BaP), indeno[*1]*, [2], [3]pyrene (InP), dibenzo[*a,h*]anthracene (DBA), and benzo[*ghi*]perylene (BgP). The 16 PAH standards and 3 deuterated PAH surrogate standards (D_10_- Acenaphthene (Ace-D_10_), D_10_Phenanthrene (Phe-D_10_), D_12_-Benzo[a] anthracene (BaA-D_12_) were purchased from Sigma. All organic solvents (HPLC grade) were purchased from Fisher Scientific. Sodium sulfate and neutral alumina were baked at 450°C for 4 h. The silica gel was cleaned with dichloromethane (DCM) before using. The neutral alumina and silica gel were activated by heating at 120°C for 12 h. Reagent grade water (5% wt.) was mixed with the neutral alumina for partial deactivation.

Chemical analysis of the crude oil followed the protocol of Liu et al. [68]. Briefly, 100 µL of crude oil was diluted to 1 mL with hexane. The sample was purified with a self-packed chromatographic column with 1 g anhydrous sodium sulfate and 8 g silica gel. The column was eluted with 50 mL dichloromethane/hexane (1∶4, v/v). The eluted solution was concentrated to 1 mL by a rotary evaporator, and preserved in a freezer (−20°C) until analysis by gas chromatography-mass spectrometry (GC/MS). Crude oil was required to be more concentrated and was analyzed again in order to determine the high molecular weight PAHs (benzo[k,j]fluoranthene, benzo[a]pyrene, indeno[1,2,3]pyrene, dibenzo[a,h]anthracene and benzo[ghi]perylene), which are at relatively low concentrations in the crude oil. Since the concentration process involved some loss of the volatile compounds, we used the concentrations of PAHs determined in the first analysis except for the high molecular weight PAHs that were estimated in the second analysis of crude oil. The composition and concentration of PAHs in the Light Louisiana Sweet Crude Oil used in these experiments are shown in the Table 2.

**Table 2:** Concentration of polycyclic aromatic hydrocarbons (PAHs, ng µL^−1^) in the crude oil used in the experiments (Louisiana light sweet crude oil).

Type of PAH	Conc. (ng µL^−1^)
*Naphthalene*	844.6
*Acenaphthylene*	85.4
*Acenaphthene*	14.0
*Fluorene*	282.3
*Phenanthrene*	608.3
*Anthracene*	8.0
*Fluoranthene*	15.3
*Pyrene*	30.8
*Benz[a]anthracene*	14.0
*Chrysene*	193.9
*Benzo[b]fluoranthene*	19.6
*Benzo[k]fluoranthene*	1.60
*Benzo[a]pyrene*	9.70
*Indeno[1,2,3-cd]pyrene*	4.37
*Dibenz[a,h]anthracene*	8.81
*Benzo[ghi]perylene*	10.95

Gelatinous zooplankton samples were freeze-dried and weighed. PAHs in gelatinous zooplankton samples were extracted by Soxhlet extractors for 24 h, using hexane and DCM (1∶1, v/v) as the extraction solution. The solution was concentrated to ca. 2 mL by a rotary evaporator and purified with a chromatographic column packed with 1 g anhydrous sodium sulfate (top), 4 g neutral alumina (middle), and 8 g silica (bottom). The concentrated solution was eluted from the column with 50 mL DCM/hexane (1∶4, v/v). The collected solution was concentrated to 0.5 mL and exchanged with hexane by a rotary evaporator. A portion of the solution was used for the PAH analysis. PAHs were analyzed using GC/MS (Shimadzu QP2010 plus) with a RXi-1MS capillary column (20 m×0.18 mm i.d., film thickness 0.18 µm). The injection volume was 1 µL sample with a split ratio of 1/20, and the helium flow was set at 0.8 mL min^−1^. The temperatures of the injector and detector were set at 260°C and 275°C, respectively. The temperature of the column was ramped from 60°C to 240°C at 10°C min^−1^, and increased to 280°C at 4°C min^−1^ and held for 3 min. Selected ion monitoring mode was used to quantify PAHs, which ranged from 126 to 279 a.m.u., and dwell time per ion was 200 ms. The average recovery of surrogate for gelatinous zooplankton was 95%. The detection limit of this method is 0.001–0.004 ng µL^−1^.

### Calculations

Mortality, as % of the incubated organisms, was estimated from the number of dead (partially or totally degraded and/or with no swimming after gently touching with a Pasteur pipette tip) individuals at the daily visual checking.

Data on mortality of gelatinous plankton larval stages versus crude oil concentration were fitted to the following sigmoid model:
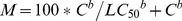
(1)where, *M* is the mortality (%), *C* is the crude oil concentration (µL L^−1^), *LC_50_* is the median lethal concentration (i.e. lethal concentration required to kill half the members of a tested population) and *b* is the slope factor.

Bioaccumulation factor is the ratio of pollutant concentration in an organism to the concentration in the ambient environment that includes dietary uptake The bioaccumulation factor (BAF) in the adult gelatinous zooplankton exposed to crude oil was calculated as follows:
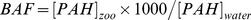
(2)where, *[PAH]_zoo_* is the concentration of polycyclic aromatic hydrocarbons (PAHs) in exposed gelatinous zooplankton after subtracting the concentration of PAHs in the corresponding control treatment, in ng g^−1^ and *[PAH]_water_* is the concentration of PAHs in seawater, in ng L^−1^ . Biomass was calculated as dry weight (DW). The concentration of PAHs in the water used in calculations were nominal concentrations estimated from the oil added to the containers, using the concentration of PAHs determined in the crude oil (Table 2).

## Results

### Toxic effects of crude oil in adult and larval stages of gelatinous plankton

We observed important differences in the sensitivity to crude oil among the tested species and life stages of gelatinous zooplankton. The scyphozoan *Pelagia noctiluca* showed 100% mortality at crude oil concentrations of 20 and 40 µL L^−1^ after 16 h (Table 3). In contrast, non-lethal effects were observed on the adult stages of scyphozoan *Aurelia aurita* at crude oil concentration ≤25 µL L^−1^ after 6 day of exposure (Table 3). Nevertheless, we observed slight tissue damage and abnormal swimming behavior in some specimens of *A. aurita* exposed to crude oil. Survival of adult stages of the ctenophore *Mnemiopsis leidyi* was not affected by crude oil at concentrations ≤5 µL L^−1^, but decreased to 79% at 25 µL L^−1^ after 6 days of exposure (Table 3). Alterations in swimming behavior (e.g., slow swimming speed) were also observed in ctenophores at the higher crude oil exposure levels.

**Table 3:** Mortality (%) of the studied species of gelatinous zooplankton (adult stages) exposed to different crude oil concentrations (1–40 µL L^−1^).

Species (Adults) 0	Crude oil conc. (µL L^−1^)					
	0	1	5	20	25	40
*Pelagia noctiluca*	0	-	-	100	-	100
*Aurelia aurita*	0	0	0	-	0	-
*Mnemiopsis leidyi*	0	0	0	-	21	-

Note that these values are the accumulative mortality after the entire incubation time (<1 day for *P. noctiluca*, 6 days for *A. aurita* and *M. leidyi*. Dash symbols indicate not determined.

**Figure 1:**
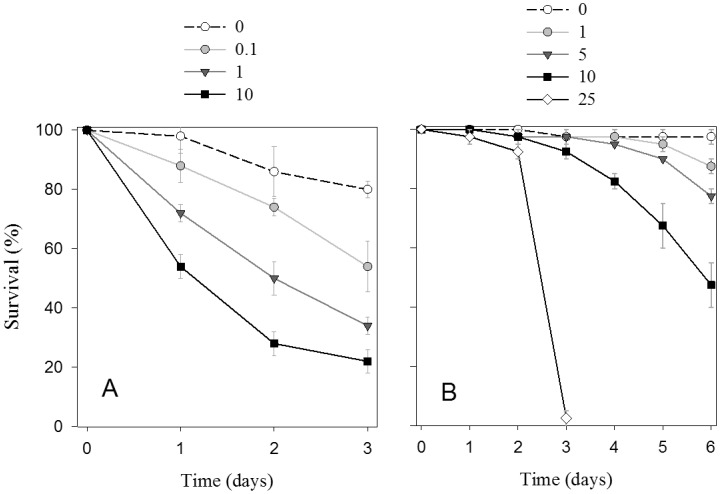
Temporal variation of the survival (%) of ephyra larvae of the scyphozoan *Aurelia aurita* (A) and cydippid larvae of the ctenophore *Mnemiopsis leidyi* (B) exposed to different concentrations of crude oil (µL L^−1^). Symbols show the average values of mortality and error bars represent the standard errors.

Survival of *Aurelia aurita* ephyra larvae and *Mnemiopsis leidyi* cydippid larvae decreased with increasing crude oil concentration and exposure time (Fig. 1). Survival of ephyra larvae decreased to less than 40% at crude oil concentrations ≥1 µL L^−1^ after 3 days of exposure (Fig. 1A). Two-way ANOVA test demonstrated significant differences in survival of ephyra larvae among crude oil exposure levels (F = 121.7, p<0.01) and exposure time (F = 70.8, p<0.01) (Fig. 1A). Lethal effects of crude oil on ctenophore cydippid larva were not observed until after 3 days of exposure, when mortality became almost 100% at the highest tested concentration, 25 µL L^−1^ (Fig. 1B). In contrast, survival of ctenophore larvae was higher than 70% at concentration ≤5 µL L^−1^ after 6 days of incubation (Fig. 1B) and differences in survival among lower crude oil concentrations (≤5 µL L^−1^) were significant only after 5 and 6 days of exposure (ANOVA: F = 12.0, p<0.05 after 5 days; F = 24.9, p<0.05 after 6 days) (Fig. 1B). Alterations in swimming behavior (e.g., low mobility, slow swimming speed) were also observed in both types of larvae when were exposed to the higher crude oil concentrations.

**Figure 2:**
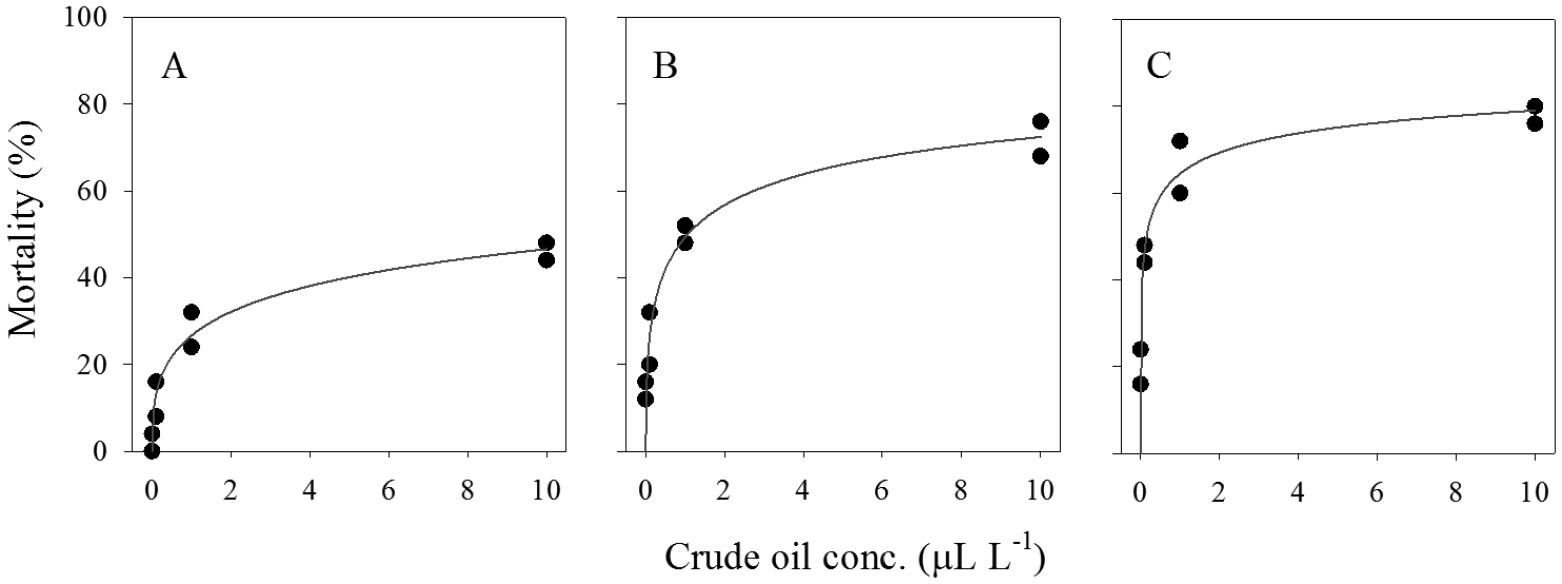
Relationships between mortality (%) of ephyra larvae of the scyphozoan *Aurelia aurita* and crude oil concentration after 24 (A), 48 (B) and 72 (C) hours of exposure. Regression lines based on Equation (1).

**Figure 3:**
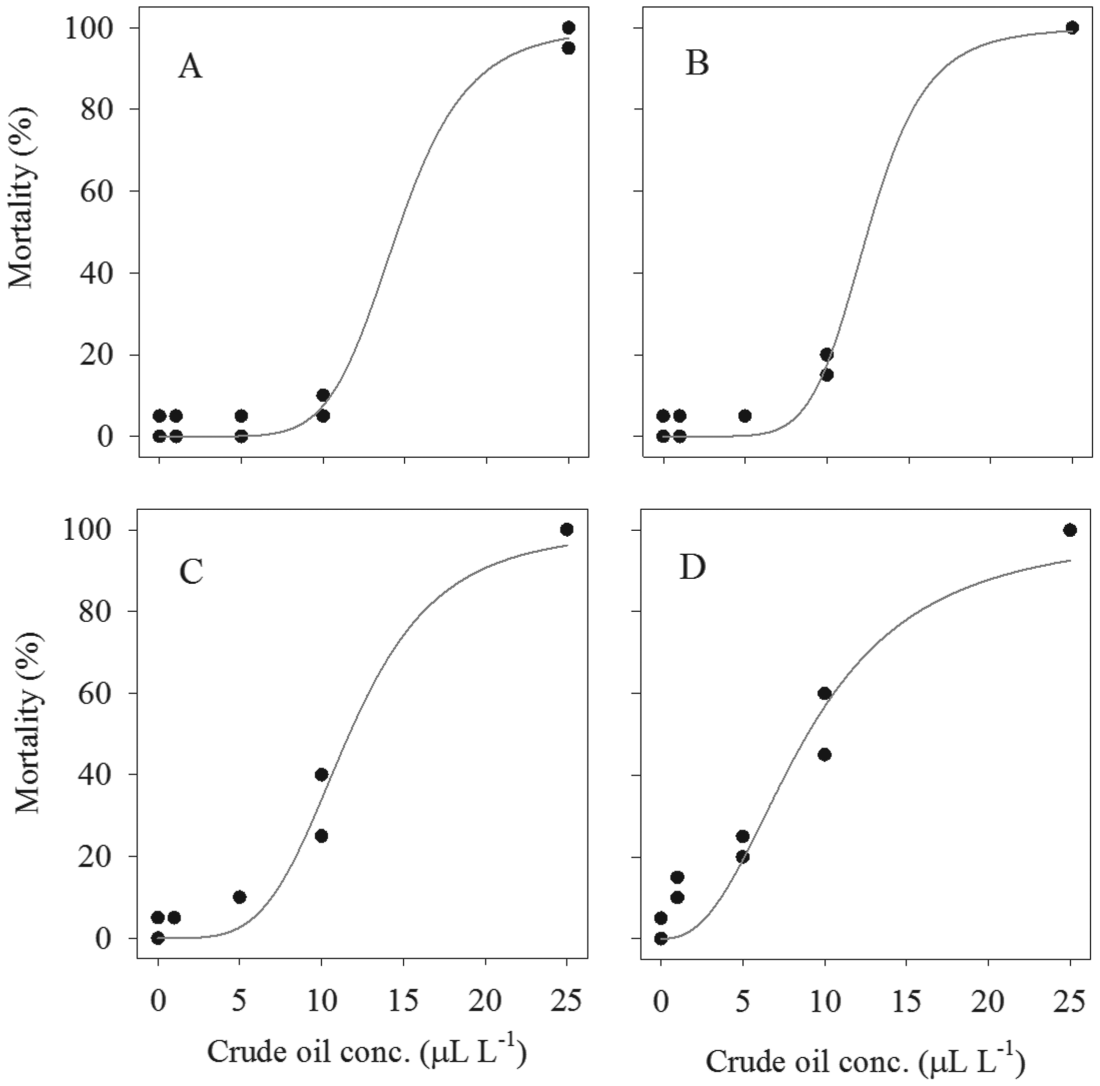
Relationships between mortality (%) of cydippid larvae of the ctenophore *Mnemiopsis leidyi* and crude oil concentration after 3 (A), 4 (B), 5(C) and 6 (D) days of exposure. Regression lines based on Equation (1).

**Figure 4:**
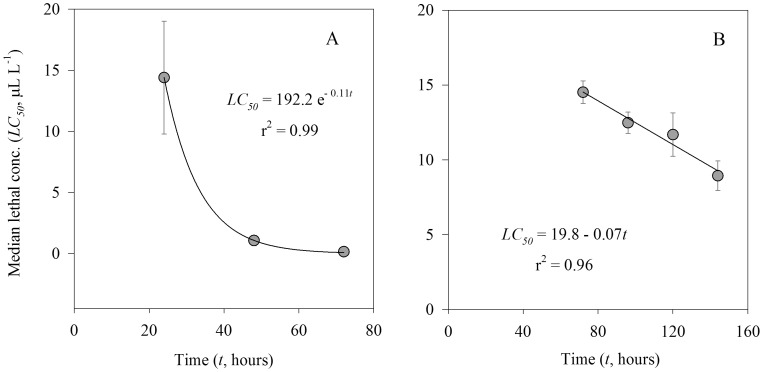
Relationships between median lethal concentration (*LC_50_*, µL L^−1^) and incubation time (*t*, hours) of ephyra larvae of the scyphozoan *Aurelia aurita* (A) and cydippid larvae of the ctenophore *Mnemiopsis leidyi* (B) exposed to crude oil.

The relationship between mortality of larval stages of gelatinous zooplankton and crude oil concentration for each incubation duration was well described by the sigmoid model (Fig. 2 & 3, Table 4). According to the model, the median lethal concentration (LC_50_) for ephyra larvae decreased from 14.41 µl L^−1^ after 1 day to 0.15 µL L^−1^ after 3 days of exposure (Table 4). The median lethal concentration for cydippid larva decreased from 14.52 µL L^−1^ after 3 days to 8.94 µL L^−1^ after 6 days of exposure to crude oil (Table 4). We observed different relationships between LC_50_ and crude oil concentration between the studied larval stages of gelatinous zooplankton (Fig. 4). LC_50_ for ephyra larvae decreased exponentially with exposure time (Fig. 4A), whereas LC_50_ for ctenophore larvae decreased linearly with exposure time (Fig. 4B).

**Table 4:** Parameters of the model (equation 1) fitted to data used to describe the relationship between mortality of gelatinous zooplankton larvae and crude oil concentration at different exposure times (Fig. 2 and 3).

Species	Stage	Exposure time (d)	LC_50_ ± ES	b ± ES	r^2^
*Aurelia aurita*	Ephyra larva	1	*14.41±4.61	*0.38±0.05	0.95
		2	*1.07±0.43	*0.43±0.10	0.87
		3	0.15±0.14	0.29±0.13	0.76
*Mnemiopsis leidyi*	Cydippid larva	3	*14.52±0.99	*6.70±1.15	0.95
		4	*12.48±1.45	*6.95±3.58	0.97
		5	*11.69±0.71	*4.22±1.17	0.99
		6	*8.94±0.76	*2.46±0.53	0.99

Note that mortality of cydippid larvae was not observed the first 2 days of exposure. LC_50_: median lethal concentration (µL L^−1^), b: shape factor, r^2^ = correlation coefficient, SD: standard deviation, ES: error standard.

* Asterisks indicate a statistical significant (p<0.05).

### Bioaccumulation of polycyclic aromatic hydrocarbons in gelatinous zooplankton

The total concentration of PAHs in the crude oil was 2.15 µg µL^−1^ (Table 1). Naphthalene, phenanthrene, fluorene, chrysene, and acenaphthylene were the most abundant PAHs in the crude oil used in our experiments (Table 1).

In crude oil exposure experiments with *Pelagia noctiluca*, the total concentration of PAHs in tissues of the experimental treatments (avg. 215 ng g^−1^ DW) was lower than in the control treatment (298 ng g^−1^ DW) because the high mortality of *P. noctiluca* (100% in 16 h) at the tested crude oil concentrations (20 and 40 µL L^−1^), and consequently bioaccumulation of PAHs was not observed.

**Figure 5:**
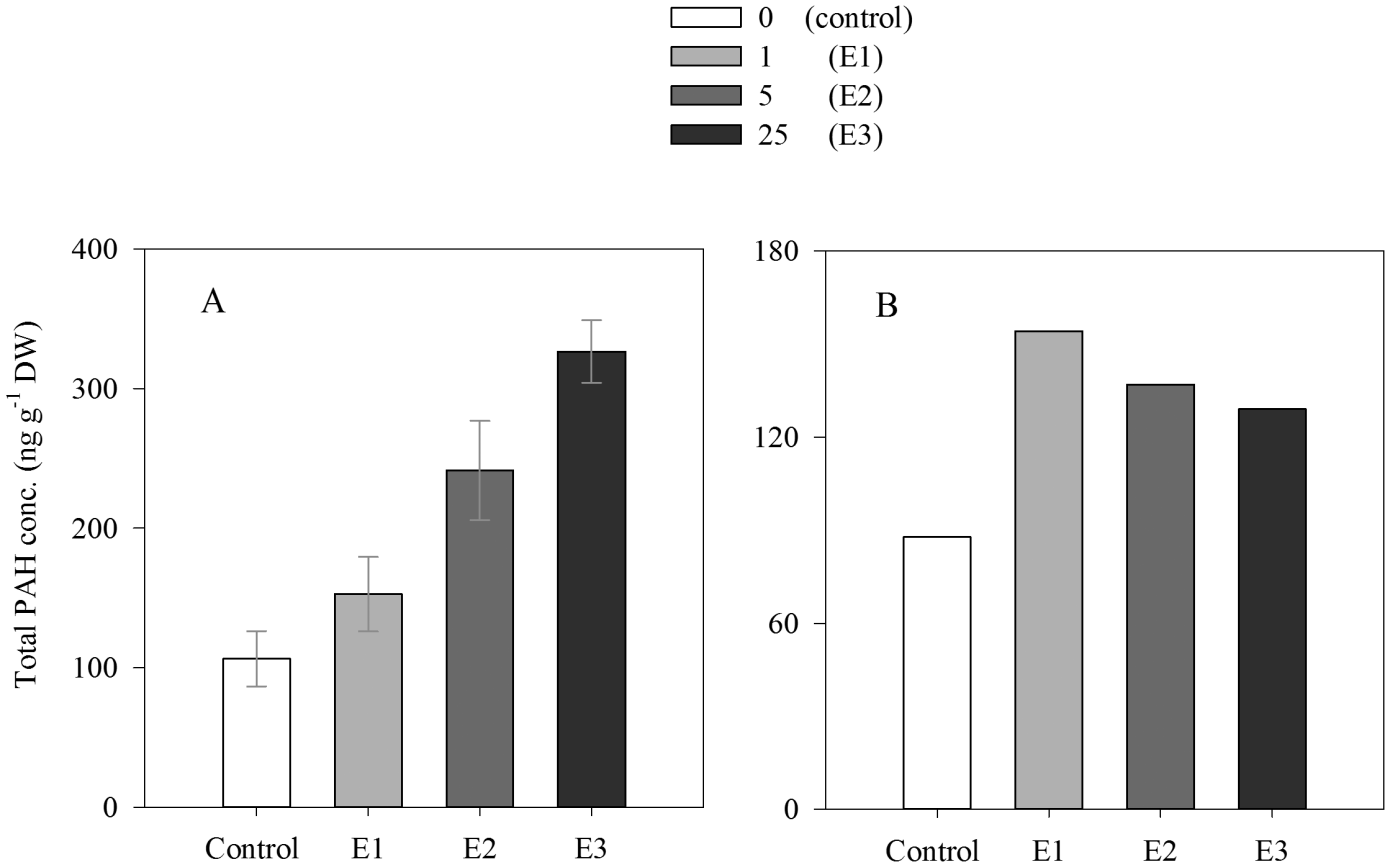
Total concentration of the polycyclic aromatic hydrocarbons (total PAHs) detected in adult stages of scyphozoan *Aurelia aurita* (A) and the ctenophore *Mnemiopsis leidyi* (B) after 6 days of exposure to different concentrations of crude oil. (Control: no oil, E1: 1 µL L^−1^, E2: 5 µL L^−1^, E3: 25 µL L^−1^.) Error bars represent the standard errors.

**Figure 6:**
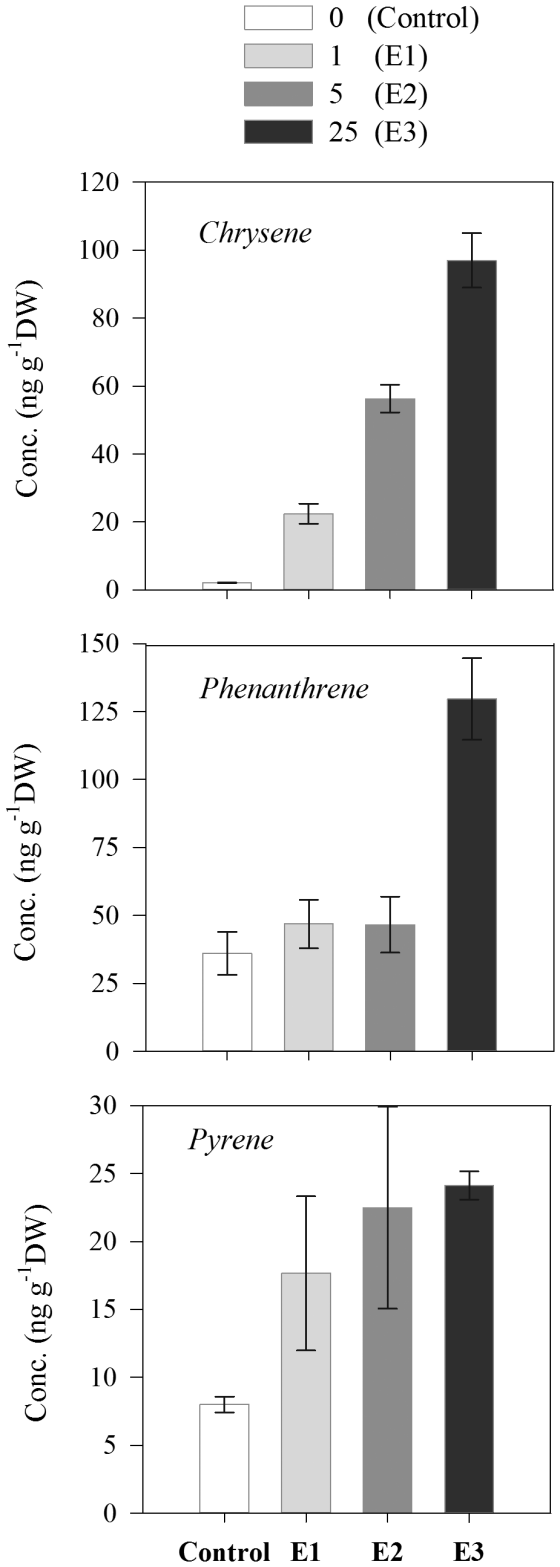
Concentration of the main polycyclic aromatic hydrocarbons detected in adult stages of scyphozoan *Aurelia aurita* after 6 days of exposure to different crude oil concentrations. (Control: no oil, E1: 1 µL L^−1^, E2: 5 µL L^−1^, E3: 25 µL L^−1^.) Error bars represent the standard errors.

Both the scyphozoan *Aurelia aurita* and ctenophore *Mnemiopsis leidyi* showed higher concentrations of total PAHs in the experimental treatments than in the controls (Fig. 5). The average total concentration of PAHs in *A. aurita* exposed to 1, 5, 25 µL L^−1^ of crude oil was 1.4, 2.3 and 3.1 times higher, respectively, than the average total concentration of PAHs in the control treatments (Fig. 5A). The total concentration of PAHs in *M. leidyi* exposed to 1, 5, 25 µL L^−1^ of crude oil was 1.8, 1.6, 1.5 times higher, respectively, than in the controls (Fig. 6). In the case of the scyphozan *A. aurita*, we observed significant differences in total concentration of PAHs among exposure levels (ANOVA, F = 14.6, p<0.05), with increasing concentration of PAHs with increasing crude oil exposure concentration (Fig. 5). In contrast, the total concentration of PAHs in *M. leidyi* decreased slightly with increased exposure levels (Fig. 5B). The concentration of total PAHs in both species was quite similar at the lowest crude oil exposure concentration (1 µL L^−1^) (Fig. 5). However, *A. aurita* showed concentrations of total PAHs 1.8 and 2.5 times higher, respectively, than *M. leidyi* at crude oil concentration exposure of 5 and 25 µl L^−1^ (Fig. 5).

Specimens of *Aurelia aurita* and *Mnemiopsis leidyi* exposed to crude oil for 10 min to evaluate if oil droplets attached to exterior of the gelatinous zooplankton bodies, showed similar or slightly lower concentrations of total PAHs (*A. aurita*: 73.4 ng g^−1^ DW in average, *M. leidyi*: 81.2 ng g^−1^ DW) to those found in the specimens in the control treatments (Fig. 5).

**Figure 7:**
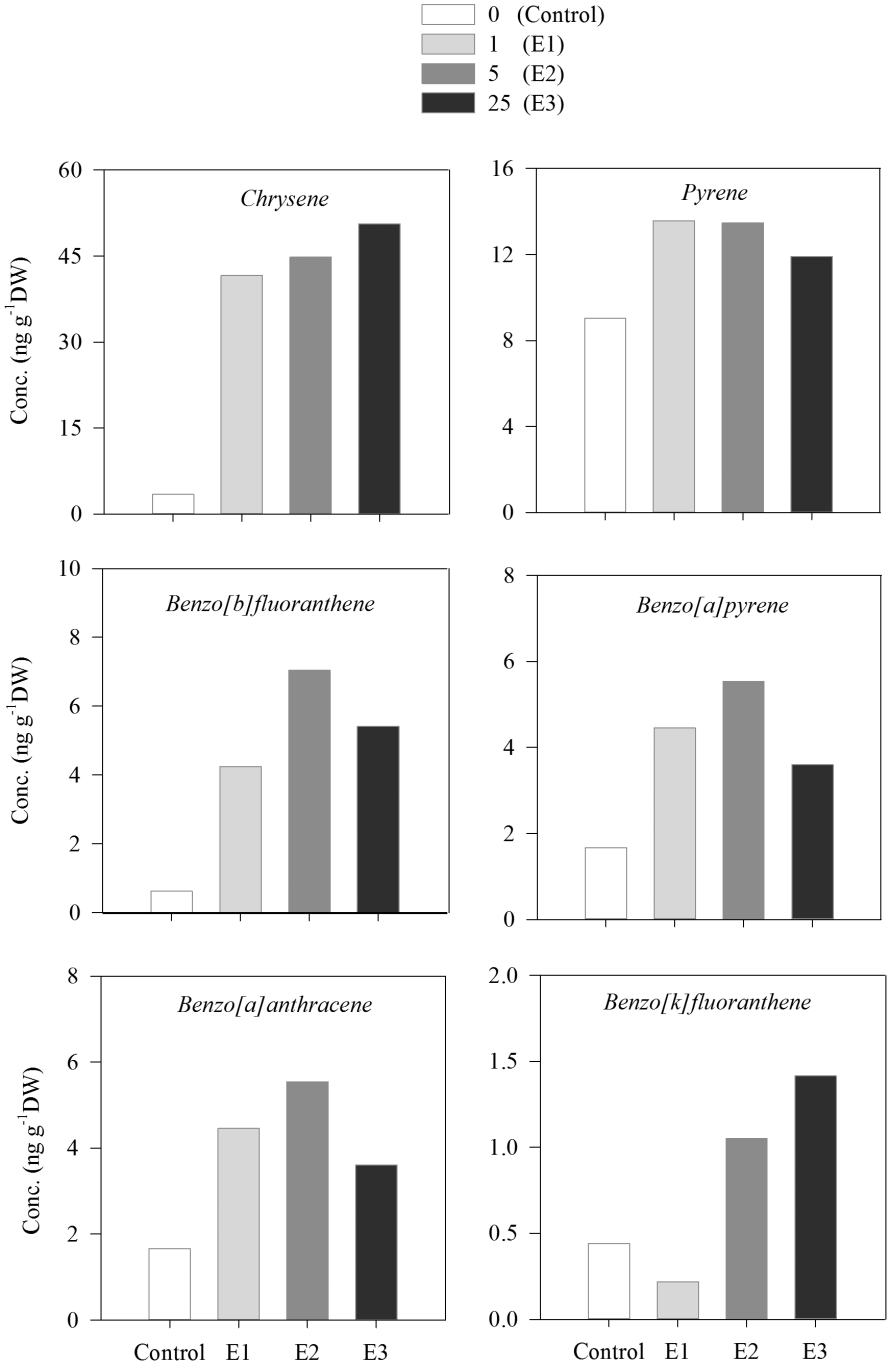
Concentration of the main polycyclic aromatic hydrocarbons (PAHs) detected in adult stages of ctenophore *Mnemiopsis leidyi* after 6 days of exposure to different crude oil concentrations. (Control: no oil, E1: 1 µL L^−1^, E2: 5 µL L^−1^, E3: 25 µL L^−1^.)

Chrysene, phenanthrene and pyrene were the main PAHs detected in *Aurelia aurita* exposed to crude oil with concentrations ranging from ca. 18 to 130 ng g^−1^ DW depending on the PAH and the crude oil exposure level (Fig. 6). Concentration of these PAHs tended to increase with increasing crude oil concentration exposure (Fig. 6). Overall, we observed a significant difference between experimental and control treatments for chrysene (ANOVA, F = 7.2, p<0.05) and pyrene (ANOVA, F = 7.9, p<0.05) (Fig. 6). For phenanthrene, significant differences between control and experimental treatments were only observed at the highest crude oil concentration exposure (ANOVA, F = 15.4, p<0.05) (Fig. 6). Chrysene, pyrene, benzo[*a*]pyrene, benzo[*b*]fluoranthene, benzo[*k*]fluoranthene, and benzo[*a*] anthracene were the main PAHs detected in *Mnemiopsis leidiy* exposed to crude oil with concentrations ranging from ca. 0.22 to 50.6 ng g^−1^ DW depending on the PAH and the crude oil exposure level (Fig. 7). Concentration of chrysene and benzo[*k*]fluoranthene tended to increase with increasing crude oil concentration exposure, whereas the concentration of other PAHs did not show a clear pattern with increasing crude oil exposure levels (Fig. 7).

Bioaccumulation factors (BAFs) ranged from 4 to 313 depending on the type of PAH, the crude oil exposure concentration and the gelatinous zooplankton species (Table 5). BAFs in *Aurelia aurita* were highest for chrysene and pyrene than for phenanthrene (Table 5). In the case of *Mnemiopsis leidyi*, BAFs were highest for chrysene, benzo[*b*]fluoranthene and benzo[*a*] anthracene than for the other detected PAHs (Table 5). In all cases, we observed a decrease in BAFs as crude oil concentration exposure increased (Table 5).

**Table 5:** Bioaccumulation factors of PAHs in gelatinous zooplankton exposed to different concentrations of crude oil (1–25 µL L^−1^).

Polycyclic aromatic hydrocarbons	*Aurelia aurita* (scyphozoan)			*Mnemiopsis leidyi* (ctenophore)		
	Crude oil exposure conc. (µL L^−1^)			Crude oil exposure conc. (µL L^−1^)		
(PAHs)	*1*	*5*	*25*	*1*	*5*	*25*
*Chrysene*	105	56	20	197	43	10
*Phenanthrene*	18	3	6	11	-	-
*Pyrene*	313	94	21	147	29	4
*Benzo[b]fluoranthene*	**-**	**-**	**-**	185	65	10
*Benzo[a]anthracene*	**-**	**-**	**-**	199	55	6
*Benzo[k]fluoranthene*	**-**	**-**	**-**	-	76	24
*Benzo[a]pyrene*	**-**	**-**	**-**	102	23	5

The hash symbol indicates that bioaccumulation was not detected (i.e., the concentration of the PAH was similar or lower than respective control treatments).

## Discussion

### Toxic effects of crude oil on gelatinous zooplankton

Evaluating the potential impact of crude oil spills on the structure and dynamics of planktonic food webs requires assessing the sensitivity of target/functional groups of zooplankton, such as gelatinous zooplankton, and their various life stages to crude oil. Toxicological studies of crude oil/petroleum hydrocarbons on gelatinous zooplankton are very scarce, making it difficult to compare results among species and to find general patterns of the effects of crude oil on this zooplankton group. Field observations and previous studies suggest that scyphozoans and ctenophores are highly tolerant to chemical water pollution and other anthropogenic impacts [41], [69]–[73]. In some polluted bays, estuaries and coastal areas there has been an increase or blooming of certain species of gelatinous zooplankton [41], [70]–[72]. For instance, increased abundance of the scyphozoan *Aurelia* has been observed in several bays and coastal areas worldwide including Tokyo and Osaka Bays, the Black Sea and the Gulf of Mexico after industrial pollution or other anthropogenic activities (e.g., oil rig construction in the Gulf of Mexico) [41], [71]. The ctenophore *Mnemiopsis leidyi* is also able to inhabit and invade polluted areas as observed in the Black and Caspian Seas [61]–[63]. According to our results and considering the median lethal concentrations of crude oil or water soluble fraction commonly observed in zooplankton, adult *Aurelia aurita* and *M. leidyi* showed much higher tolerance to crude oil or petroleum hydrocarbons than other zooplankton, such as copepods [8], [16], [31], [75]–[78], fish larvae [79]–[80], and other invertebrates [81]–[83]. A previous study found that the median lethal concentration for adult *M. leidyi* after 3 days of exposure to the water soluble fraction of crude oil was 3.3 mL L^−1^ [84], which is an extremely high, unrealistic concentration considering the typical concentrations observed in seawater after oil spills [85]–[87]. Overall, our results confirm that the adult scyphozoan *A. aurita* and ctenophore *M. leidyi* are highly tolerant to crude oil pollution, which may partially explain their enhanced capacity to inhabit and increase their abundance in polluted coastal habitats.

It is important to note that coastal pollution has also been frequently associated with a loss of the diversity in gelatinous plankton (e.g., decrease in hydromedusa species) [41], [71]. Only certain species of gelatinous zooplankton from bays, estuaries and semi-open coastal areas (e.g., *Aurelia aurita*, *Mnemiopsis leidyi*, *Rhizostoma* sp., *Chrysaora* sp.) show a high tolerance to crude oil or other types of anthropogenic pollution [69], [74]. In contrast, little is known about the effects of oil pollution on offshore pelagic species of gelatinous zooplankton. In our study, we observed that small-sized adult stages of *Pelagia noctiluca*, a typically offshore species, were highly sensitive to crude oil compared to the adult scyphozoan *A. aurita* and ctenophore *M. leidyi*, and with other zooplankton groups [8], [75]–[80]. Hence, although generalizations should be considered carefully due to the limited information, our results suggest that gelatinous zooplankton from estuaries and coasts have a higher tolerance to crude oil pollution than offshore oceanic species, such as *P. noctiluca*. This conclusion agrees with previous studies on crustacean zooplankton that found coastal zooplankton tend to be more tolerant to petroleum hydrocarbons than offshore oceanic zooplankton [76]. Therefore, although some species are highly resistant to oil pollution, other species or groups of gelatinous zooplankton (e.g., the scyphozoan *P. noctiluca*, hydromedusae) may be more susceptible to be negatively impacted by oil spills, which may affect marine food web interactions mediated by these species of gelatinous zooplankton.

Although broad generalizations on the differences in sensitivity to crude oil depending on life stage should be avoided, it has been commonly observed than larval stages of invertebrates and fish are more sensitive to oil pollution than adults, with some exceptions [88]–[90]. Our results also showed that larval stages of gelatinous zooplankton were much more sensitive to crude oil exposure than adult stages, with ephyra larvae of scyphozoan *Aurelia aurita* being the most negatively affected stage. Previous studies have found that exposure to crude oil and certain petroleum hydrocarbons produces morphological abnormalities in ephyra larvae of *Aurelia* [91]. Hence, crude oil may negatively affect *A. aurita* during early development, and consequently, affect recruitment and population dynamics of this species in areas contaminated by petroleum hydrocarbons. However, the *Mnemiopsis leidyi* cydippid larvae showed a higher tolerance to crude oil exposure, suggesting that this species may be able to complete their development and life cycle at relatively high crude oil exposure concentrations, providing an adaptive advantage to inhabit and invade oil polluted coastal areas compared to *A. aurita*. More studies on the effects of crude oil in other life stages, e.g., planula larvae in *A. aurita* and early embryos in *M. leidyi*, are required to better evaluate of the impact of oil pollution in recruitment and population dynamics of these species of gelatinous zooplankton.

Toxicity of crude oil to zooplankton is strongly related to its chemical composition. Crude oil is a complex mixture of both hydrocarbons, such as alkanes, cycloalkanes and aromatic hydrocarbons, and non-hydrocarbon compounds [1]. Among petroleum hydrocarbons, polycyclic aromatic hydrocarbons (PAHs) are considered to be the most acutely toxic components. PAHs exert their toxicity by interfering with the function of cellular membranes (membrane fluidity) and with enzyme systems associated with the membrane [92]. PAHs are also associated with potential carcinogenic, teratogenic and mutagenic effects to aquatic animals and humans [93]–[96]. For gelatinous zooplankton, it has been observed that exposure to petroleum hydrocarbons result in teratological effects and possibly somatic mutations in the scyphozoan *Aurelia* sp. [91]. Besides these adverse effects, previous studies have found sublethal effects of petroleum in gelatinous zooplankton, including cessation of feeding in the pelagic tunicate *Dolioletta gegenbauri* at crude oil concentrations of 31 mg L^−1^ after 4 h [18] and abnormal swimming behavior in ephyra larvae exposed to dissolved petroleum hydrocarbons [91]. In agreement with the last finding, we also observed abnormalities in the swimming behavior (e.g., slow speed, inverse swimming, low mobility, etc.) of larvae and adults of *Aurelia aurita* and *Mnemiopsis leidyi*, at the higher crude oil exposure levels (5 and 25 µL L^−1^). If these sublethal effects are prolonged and not reversible, it may affect vital physiological activities (e.g. feeding) and consequently cause death, or may increase the risk of mortality by predation in nature.

In the natural environment, impacts of oil spills on zooplankton depend on many physical, chemical and biological factors, and therefore the effects of oil pollution on zooplankton, including gelatinous zooplankton, vary depending on the circumstances of each spill [97]. Many variables, such as the type of oil, the use of chemical dispersant, and the weathering process may affect the toxicity of crude oil to marine zooplankton after oil spills. For instance, the type of crude oil used in these experiments (Louisiana light sweet crude) is considered less toxic than other types of crude oils (e.g., N.2 Fuel Oil, Bunker C oil) and refined oils due to its lower concentrations of PAHs [1]. A typical crude oil may contain 0.2 to >7% total PAHs [1]. Considering a crude oil density of 0.845 g mL^−1^, the percent of total PAHs in crude oil used in our experiments would be 0.25%. This concentration of PAHs is expected for light crude oils, like Louisiana light sweet crude oil, which typically have lower concentrations of PAHs than heavy crude oils [1]. Similarly, although there is no available data for gelatinous zooplankton, crude oil treated with chemical dispersant could be more toxic than oil alone to gelatinous zooplankton as observed in other zooplankton groups [8], [98]–[100]. On the other hand, weathered oil is generally less toxic than fresh crude oil [1], [101]. In open marine systems with strong winds and breaking surface waves, some of the toxic compounds of the crude oil, such as the monoaromatic hydrocarbons (benzene, toluene, ethyl benzene and xylenes), may be lost by evaporation, reducing the potential toxicity of oil after several days [1]. In our study, experiments with larvae were conducted in closed containers and therefore we assume little or no loss of volatile fraction of crude oil; whereas in the experiments with the adult stages, although the aquarium were covered, they could have some loss of the volatile compounds of crude oil. In general, acute toxicity increases as the molecular weight increase and monoaromatic hydrocarbons are considered the least toxic of the petroleum aromatic compounds [82], [102], [103]. Since the crude oil was renewed daily, and that dissolved aromatic hydrocarbons are the most toxic compounds to marine organisms [82], [102], [103], we considered the loss of some volatile fraction in the aquariums to have had a low influence on our conclusions about the acute toxicity of crude oil in gelatinous zooplankton. However, more research is required to determine the differences in the toxicity between fresh crude oil and weathered oil, and the different compounds of crude oil to gelatinous zooplankton.

Oil toxicity may also vary widely depending on environmental variables, including temperature [29], salinity [104], light [105], [106], and turbulence [107]. Among the different extrinsic variables affecting oil toxicity, UV radiation (UVR) seems to play an important role in the toxicity of crude oil to zooplankton [108]–[111]. Previous studies have shown UVR may increase the toxicity of petroleum by 2- to 50,000-fold due to the photosensitization and/or photomodification of the polycyclic aromatic hydrocarbons [108]–[111]. Gelatinous zooplankton would be particularly vulnerable to the photoenhanced toxicity of crude oil because most of these organisms are translucent/transparent and frequently are adapted to live in the upper layers of the water column (neuston) and in shallow coastal areas with elevated UVR. Therefore, more studies about the effect of crude oil on zooplankton with different environmental conditions, particularly with natural sunlight exposure (UVR), are required for a better assessment of the impact of crude oil spills in gelatinous zooplankton.

### Bioaccumulation of polycyclic aromatic hydrocarbons in gelatinous zooplankton

Gelatinous zooplankton may take up petroleum hydrocarbons directly, through passive uptake (cutaneous absorption of dissolved petroleum hydrocarbons) or ingestion of oil droplets, and/or indirectly, through the ingestion of contaminated zooplankton and/or phytoplankton. Information on the uptake and bioaccumulation of petroleum hydrocarbons by gelatinous zooplankton is very limited. Lee (1975) reported that the ctenophore *Pleurobrachia pileus*, and an unidentified “jellyfish” species rapidly took up and accumulated certain dissolved polycyclic aromatic hydrocarbons (e.g., benzopyrene) from seawater [112]. In that study, petroleum hydrocarbons were detected in ctenophores after being fed with copepods labeled with ^3^H-benzopyrene, indicating uptake of petroleum hydrocarbons by the dietary route [108].We also found that the scyphozoan *Aurelia aurita* and the ctenophore *Mnemiopsis leidyi* accumulated certain petroleum hydrocarbons, including benzopyrene in the case of *M. leidyi*. A recent study found that the pelagic tunicate *Dolioletta gegenbauri* ingested and defecated small dispersed oil droplets (1–30 µm in diameter) [18]. Overall, these results suggest that gelatinous zooplankton may play a role in the fate of crude oil in the sea after oil spills.

Most crude oil toxicity and PAH bioaccumulation studies on zooplankton, including gelatinous zooplankton, have been conducted using the crude oil water soluble fraction (WSF), or certain mixed or individual PAHs. However, since some gelatinous zooplankton can ingest oil droplets [18], exposure to dispersed crude oil may promote the uptake of PAHs as compared with experiments using WSF, as observed in fish [113]. In our experiments, since we used crude oil emulsions instead of WSF, it is possible that oil droplets could attach externally to the body of gelatinous zooplankton, which has been observed in laboratory and field studies in other zooplankton groups [12]. However, the use of filtration and high pressure washing substantially removed any attached oil droplets, as corroborated by the analysis of samples of specimens exposed for 10 min to crude oil using this methodology, which have similar concentrations of total petroleum hydrocarbons as the controls. Hence, the external attachment of oil droplets to the body of the animals did not contribute to the bioaccumulation of petroleum hydrocarbons detected in gelatinous zooplankton in our experiments. Nevertheless, in the natural environment, the adhesion of crude oil droplets to gelatinous zooplankton after oil spills may be another route of transfer of PAHs up through marine food webs. It is important to note that, in contrast to our experiments, many acute toxicological and bioaccumulation studies with zooplankton, including gelatinous zooplankton, have been conducted without food [30], [89], [114]–[115]. However, as mentioned before, the dietary intake of petroleum hydrocarbons may be more relevant for gelatinous zooplankton because crustacean zooplankton/phytoplankton may accumulate higher concentrations of PAHs than gelatinous zooplankton [21] and the bioaccumulation factor of some petroleum hydrocarbons ingested through the diet may be higher than from the dissolved state in seawater [116]. Moreover, some gelatinous zooplankton, such as pelagic tunicates, as well as other zooplankton (e.g. protozoa) only ingest oil droplets in the presence of food, e.g. phytoplankton [13], [18]. Therefore, starvation conditions in petroleum exposure experiments may lead to important bias in the quantification of the potential uptake and bioaccumulation of petroleum hydrocarbons by zooplankton.

Bioaccumulation of PAHs in zooplankton varies widely depending on the species/groups of zooplankton and the experimental approach [8], [12], [21], [73], [110]–[112], [117]. We observed important quantitative and qualitative differences in bioaccumulation of petroleum hydrocarbons after exposure to crude oil between the two studied species of gelatinous zooplankton and also comparing gelatinous zooplankton with crustacean mesozooplankton [8]. Concentration of total PAHs (ng g^−1^ DW) in *Aurelia aurita* was higher than in the ctenophore *Mnemiopsis leidyi* when exposed to crude oil concentration ≥5 µl L^−1^. Both species showed total concentration of PAHs per biomass of dry weight one order of magnitude lower than those observed in crustacean mesozooplankton communities exposed to similar types and concentrations of crude oil [8]. These important quantitative differences in bioaccumulation may be partly related to differences in biochemical composition among these species/zooplankton groups, particularly their lipid content. PAHs are lipophilic and are usually accumulated in the lipids of organisms. Although lipid content is highly variable among species and groups of zooplankton, gelatinous zooplankton frequently have lipid content by dry weight that is an order of magnitude lower than crustacean zooplankton [118]–[122] and within gelatinous zooplankton, scyphozoans (medusa) generally have more lipids than ctenophores [119].

Besides the differences in the amount of total PAH accumulated between the studied species of gelatinous zooplankton, we observed a selective bioaccumulation of petroleum hydrocarbons, i.e. accumulation of only certain PAHs, with differences between the species/zooplankton groups. According to our results, although gelatinous zooplankton accumulate less petroleum hydrocarbons than crustacean zooplankton [8], they tend to accumulate mainly PAHs with high molecular weight, which are considered more toxic than the low molecular weight PAHs. In fact, some of the PAHs accumulated in the ctenophore *Mnemiopsis leidyi* e.g. benzo[*a*]pyrene, benzo[*b*]fluoranthene, benzo[*k*]fluoranthene, and benzo[*a*]anthracene, are considered the most toxic/harmful components of crude oil, with potential carcinogenic, teratogenic and mutagenic effects to aquatic animals and humans [93]–[95]. When uptake and removal of petroleum hydrocarbons is due to passive partitioning alone, BAF of PAHs are associated with their lipophilic properties, i.e., octanol–water partition coefficient, K_ow_, with log BAF increasing linearly with log K_ow_ [123]–[124]. This pattern has been commonly observed in acute tests conducted with zooplankton exposed to some specific dissolved PAH or the water-soluble fraction (WSF) of crude oil [21], [30]. We also found BAF tended to be lower for PAH with low K_ow_ (i.e., phenanthrene), than for PAH with higher K_ow_ (i.e., pyrene, chrysene, benzo[*b*]fluoranthrene, benzo[*a*]anthracene). Since we used crude oil instead of dissolved petroleum hydrocarbons, the deviations from the linear relationship between log BCF and log K_ow_ observed in our studies may be due to the lower availability of more hydrophobic compounds in the water and the ingestion of oil droplets or contaminated prey. Therefore, comparison between BAF of petroleum hydrocarbons using crude oil instead of WSF should be done cautiously.

Besides the chemical properties of PAHs and the lipid content of the animal, bioaccumulation of petroleum hydrocarbons in marine animals is inversely related to the capacity of the organisms to depurate petroleum hydrocarbons by excretion or egestion, or other physiological mechanisms [21], [115], [125], [126]. Lee (1975) found that petroleum hydrocarbons (e.g., benzo[a]pyrene) were metabolized to more polar metabolites by crustaceans but not by ctenophores or jellyfish, although discharge of ingested petroleum hydrocarbon also occurred in the gelatinous zooplankton species [112]. This result suggests that some gelatinous zooplankton species may have a limited capacity to depurate PAHs, which implies that PAHs may reside in the tissues of gelatinous zooplankton for longer, thus increasing the possibility of these toxic compounds to be transferred up the food web. Unfortunately, little is known about the depuration mechanisms of gelatinous zooplankton and more studies are required to determine how the ability to metabolize petroleum hydrocarbons and time required for depuration differs among target zooplankton groups.

In light of our results, research on the toxic effects of crude oil on gelatinous zooplankton, including the potential role of these zooplankton in the bioaccumulation and biotransfer of PAHs after oil spills, should receive more attention considering that >100 species of fish, including commercial species (e.g., Atlantic Bluefin tuna, *Thunnus thynnus*; chum salmon *Oncorhynchus keta*), sea turtles (leatherback sea turtle, *Dermochelys coriacea*), and dozens of other animals feed on gelatinous zooplankton [52]–[54], [74]. Further, some of these species of top consumers feed almost exclusively on gelatinous zooplankton (e.g. ocean sunfish, *Mola mola*; leatherback sea turtle, *Dermochelys coriacea*) [52]–[54], [74]. Therefore, toxic effects of crude oil on gelatinous zooplankton, e.g. decrease in abundance of offshore species, may affect the population of these fish and apex predator species. Moreover, consequences of ingesting contaminated gelatinous zooplankton to these top consumers are unknown. Field and laboratory studies have shown that, although the elimination of PAHs is generally efficient in vertebrates (e.g. fish), the metabolism of PAHs not only results in detoxication but can also induce histopathological lesions and generate genotoxic metabolites [127]–[130]. Understanding the toxic effects and bioaccumulation of PAHs in gelatinous zooplankton after oil spills is particularly important in the Gulf of Mexico because it is a spawning area for many species of migrant pelagic fish and sea turtles that feed on gelatinous plankton in these waters. Consequently, any negative impact of an oil spill mediated by gelatinous zooplankton in the Gulf of Mexico would affect the global populations of these important species. Since some gelatinous zooplankton show high tolerance to crude oil, and can accumulate very toxic PAHs, presumably with a lower depuration capacity compared to other zooplankton , we highly recommend the use of gelatinous zooplankton (e.g. scyphozoans and ctenophores), together with other relevant zooplankton groups (e.g., copepods), as biomonitors/bioindicators of petroleum hydrocarbon pollution after oil spills. Overall, although gelatinous zooplankton have been previously ignored, our results indicate that knowledge of the interactions between gelatinous zooplankton and crude oil is necessary to better understand the fate of petroleum hydrocarbons in the pelagic zone after oil spills and the impact of crude oil pollution on the marine environment.
